# Breastfeeding practices based on the gestational age and weight at birth in the first six months of life in a population-based cohort of infants from North India

**DOI:** 10.3389/fped.2023.1127885

**Published:** 2023-06-26

**Authors:** Sitanshi Sharma, Ranadip Chowdhury, Sunita Taneja, Sarmila Mazumder, Kiran Bhatia, Runa Ghosh, Sowmya C. Karantha, Neeta Dhabhai, Harish Chellani, Rajiv Bahl, Nita Bhandari

**Affiliations:** ^1^ Centre for Health Research and Development, Society for Applied Studies, New Delhi, India; ^2^ Department of Pediatrics, Vardhman Mahavir Medical College and Safdarjung Hospital, New Delhi, India; ^3^ Department of Maternal, Newborn, Child and Adolescent Health, World Health Organization, Geneva, Switzerland

**Keywords:** preterm, SGA, gestational age (GA), birth weight, breastfeeding, exclusive breast feeding (EBF)

## Abstract

**Background:**

Short and long term benefits of early Initiation of breastfeeding (EIBF) and exclusive breastfeeding (EBF) in the first six months of life are well established and recommended globally. However, reliable estimates of breastfeeding practices and impact of breastfeeding counselling interventions according to gestational age and weight at birth are not available in low and middle income countries.

**Objective:**

To assess the impact of breastfeeding counselling on EIBF and EBF during the first 6 months of life according to gestational age and weight at birth.

**Methods:**

We analysed the data collected from the Women and Infants Integrated Interventions for Growth Study (WINGS), an individually randomized factorial design trial. Mothers were counselled on EIBF during third trimester of pregnancy. They were supported throughout the first 6 months to continue EBF by early problem identification, frequent home visits and assistance in expressing breastmilk when direct breastfeeding was not possible. Breastfeeding practices were ascertained through 24 h recalls at infant ages 1, 3 and 5 months for both the intervention and control groups by an independent outcome ascertainment team. The World Health Organization (WHO) definitions were used for classification of infant breastfeeding practices. Generalized linear models of the Poisson family with a log-link function were used to estimate the effect of interventions on breastfeeding practices. The relative measures of effect on breastfeeding practices were estimated in term appropriate for gestational age (T-AGA), term small for gestational age (T-SGA), preterm AGA (PT-AGA), preterm SGA (PT-SGA) infants.

**Results:**

Amongst all infants irrespective of gestational age and weight at birth, EIBF was (51.7%) higher amongst the intervention group (IRR 1.38, 95% CI 1.28–1.48) compared with the control group. The proportion of exclusively breastfed infants at ages 1 month (IRR 1.37, 95% CI 1.28–1.48), 3 months (IRR 2.13, 95% CI 1.30–1.44) and 5 months (IRR 2.78, 95% CI 2.58–3.00) were higher in intervention group than control group. We identified significant interaction (*p* value for interaction <0.05) between intervention and infant size and gestation at birth on exclusive breastfeeding at 3 and 5 months of age. Subgroup analysis showed that the impact of the intervention was greater on exclusive breastfeeding in PT- SGA infants at 3 months (IRR 3.30, 95% CI 2.20–4.96) and 5 months of age (IRR 5.26, 95% CI 2.98–9.28).

**Conclusion:**

This is one of the first studies wherein impact of breastfeeding counselling interventions in the first 6 months of life was assessed according to infant size and gestation at birth wherein gestational age was reliably estimated. The impact of this intervention was higher in preterm and SGA babies compared to other infants. This finding is important as preterm and SGA infants have a higher burden of mortality and morbidity during early infancy. Intensive breastfeeding counselling to these vulnerable infants is likely to improve overall breastfeeding rates and reduce the adverse outcomes.

**Clinical Trial Registration**: [http://ctri.nic.in/Clinicaltrials/pmaindet2.php?trialid=19339%26EncHid=%26userName=societyforappliedstudies], identifier [#CTRI/2017/06/008908].

## Introduction

Exclusive breastfeeding (EBF) during the first 6 months of life is universally recommended as it reduces the risk of neonatal and childhood morbidity and mortality. EBF supports optimal growth, neurodevelopment and better school performance (1). Furthermore, evidence from literature supports maternal benefits for breastfeeding like reduced risk of type 2 diabetes mellitus, prolonging lactation amenorrhoea and protection from various cancers like ovarian and breast cancers (2).

Despite the evidence, EBF rates continue to be sub-optimal in the low-middle income countries (LMIC’s) (3). Promotion of EBF for first 6 months of life is a part of all national and international guidelines. Breastfeeding practices of vulnerable babies like preterm and small for gestational age (SGA) babies are not reflected in demographic surveys (4).

Babies born preterm (born before completing 37 weeks of gestation) and small for gestational age (born with birth weight <10th centile for gestational age at birth) are vulnerable to serious infections and feeding difficulties (5). This contributes to increased risk of growth failure, death in early and later life, and neurodevelopmental deficits (6, 7). Breastmilk provides protection for preterm and SGA infants by reducing severity of necrotizing enterocolitis, sepsis, and retinopathy of pre- maturity. Breastfeeding also improves neuropsychological performance, strengthens mother-child bond, reduces the length of hospital stay with lesser incidence of readmissions (8). In spite of the evidence on benefits of breastfeeding for preterm and small for gestational age (SGA) babies, the knowledge on their breastfeeding practices is scanty in LMICs when compared with term infants (9, 10). The data available on preterm births is robust in high income countries with higher birth registration coverage for preterm births in contrast to LMIC’s, where birth registration coverage is low and data on preterm birth is scarce (11). Though, birthweight continues to be the primary measure for birth outcomes, it does not help in differentiating between growth restricted or preterm infants. Gestational age is a better indicator but difficult to ascertain accurately, in low resource settings (12).

We conducted a secondary analysis from a population-based cohort in urban and peri-urban low-to-mid socio-economic neighbourhoods of South Delhi to estimate the impact of breastfeeding counselling on early initiation of breastfeeding (EIBF) and EBF during the first 6 months of life for infants according to both gestational age and weight at birth. We had reliable and early estimates of gestational age by dating ultrasounds and reliable measure of birth weights to be able to categorise infants in the groups: term appropriate for gestational age (T-AGA), term small for gestational age (T-SGA), preterm AGA (PT-AGA), preterm SGA (PT-SGA) infants.

## Methods

### Study design and setting

Data collected from a recently concluded randomized controlled study, Women and Infants Integrated Interventions for Growth Study (WINGS) was used (13). Total live births which occurred during the course of the study were included in this analysis. The infants who were born to mothers who received interventions during pregnancy continued to receive the interventions throughout early childhood, and those infants who were born to mothers who were in control group during pregnancy were part of the control group during early childhood. The women underwent randomisation twice during the study due to the factorial design of the trial. The first randomisation was done when eligible women were identified through a door-to-door survey and enrolled after getting a written consent. The second randomisation was done at the time when pregnancy was confirmed. Details on the main study design and randomisation have been previously published (14). The gestational age was assessed between 9 and 13 weeks of gestation using Intergrowth-21st standards by calculating fetal crown-rump length (CRL), if CRL was >95 mm, femur length and head circumference was be used to assess gestational age (14–16). Birth weight was taken at the place of birth or at home at age day 7 (±6) days after birth by pair of trained and standardised study team workers using calibrated digital weighing scale (model 354; Seca, California, USA) to the nearest 10 gram.

Women in the intervention group received breastfeeding support which was initiated as soon as the woman reached the third trimester of pregnancy. Pregnant women were counselled on benefits of EIBF and EBF at each contact with the study team. Post birth, the study team supported mothers in EIBF within first hour of birth or as early as possible if birth occurred at the collaborating hospital. Home visits were made by study workers who were referred to as *Prernas* (inspiration) for the mothers in intervention group for all infants on days 3, 7, 10, 14, 28 after birth and thereafter monthly from 2 to 6 months of life to enquire about infant wellbeing, establish breastfeeding and promoting EBF for first 6 months (13, 14, 17). During these visits, mothers were counselled and supported for establishing and sustaining exclusive breastfeeding during the first 6 months of infant’s life. Extra support through additional home visits was given by workers trained in breastfeeding support called lactation counsellors. The lactation counsellors supported those mothers who reported breastfeeding problems to Prernas. They addressed breastfeeding problems like inverted nipple and breast engorgement or helped mothers with preterm babies. In addition, breast pumps for expressing breastmilk were provided to the mothers of small babies (mostly preterm or SGA) who were unable to suckle effectively and for some mothers with breast problems. Repeat visit were made whenever required or requested by mothers. Monthly anthropometric measures for routine growth monitoring was taken by an independent team, to observe infant growth. When inadequate weight gain (<15th centile of weight/velocity per month from birth to 6 months) was reported, the infants were referred to physicians for evaluation (18). Nutritional supplementation included vitamin D (400 IU), to all infants and Iron supplementation was given from two weeks for the very low birth weight (VLBW) and from six weeks to low birth weight (LBW) infants until six months of age according to WHO guidelines (14, 19). Snacks, milk (600 kcal, 20 g protein), micronutrient supplements, iron folic acid, calcium, and vitamin D were given to mothers for six months to meet additional requirements during lactation. In addition, counselling on positive thinking, screening and management of depressive symptoms, and WaSH interventions were provided to all mothers in intervention group (14, 20–22).

In the control group, during pregnancy, women were encouraged to register for antenatal care at a government or private facility, have at least four antenatal care check- ups, consume iron folic acid, calcium, vitamin D daily throughout pregnancy, access supplementary foods through the Integrated Child Development Services (ICDS) scheme and deliver in health facilities. After child birth, mothers were advised to go for a postnatal health check-up, and to consume iron folic acid, calcium, vitamin D, and supplementary foods daily through the ICDS scheme. They were also encouraged to allow home visits by the health workers from national health system like ASHA workers in the first 42 days of life. For early childhood, mothers were advised to breastfeed their babies exclusively for the first six months, and continue breastfeeding for at least two years and to collect supplementary food from ICDS (13).

### Outcome assessment

Outcomes were assessed at birth and at infant ages 1, 3 and 5 months. These included rates of EIBF, EBF and breastfeeding practices. An independent outcome assessment team collected data on breastfeeding practices at birth (within 7 days of birth) and at 1,3, 5 months (150–170 days) of age through 24-hour recall from the mothers.

### Definitions

Birth weight was defined as weight taken by the study team at day 7 (±6 days) after birth.

T-AGA was defined as gestational age ≥37 weeks at birth and birth weight ≥10th centile to ≤90th centile; T-SGA: gestational age ≥37 weeks at birth and birth weight <10th centile; PT-AGA: gestational age <37 weeks at birth and birth weight ≥10th centile to ≤90th centile; PT- SGA: gestational age <37 weeks at birth and birth weight <10th centile, all in accordance to the Intergrowth-21st standards (15). Sub-group analysis included those infants whose gestational age and birth weight were available.

All breastfeeding practices were defined using WHO definitions (23, 24). Early Initiation of breastfeeding was defined as initiation of breastfeeding the infant within 1 h of birth. Exclusive breastfeeding was defined as feeding the infant only breast milk (including expressed breast milk) and no other food or drink, not even water, for first 6 months of life, other than ORS, vitamins, minerals and medicines.

Predominant breastfeeding was defined as feeding the infant breast milk (including expressed breastmilk). However, the infant may also have received other liquids like water and water-based drinks, fruit juice, ritual fluids or any other liquids.

Partial breastfeeding was defined as giving the infant some breastfeeds along with either packaged or powdered milk or cereal based feeds, or any food other than breastmilk.

No breastfeeding was defined as, any infant who did not receive either direct or expressed breastmilk (23, 24).

### Statistical analysis

Data collection for the outcomes ended on 30th June 2021. For this analysis, we compared the mothers and infants received interventions during pregnancy and early childhood with the group who did not receive the interventions during that period. We assessed similarity of proportions of baseline characteristics across groups to check for successful randomisations. Intention to treat analysis was used. We used generalised linear models of the Poisson family with a log link function to estimate the effect (Incidence rate ration IRR, 95% CI) of intervention on EIBF and EBF at1, 3 and 5 months of age. The final models were adjusted for place of birth, family possessing a below poverty line card, women’s height, and women’s body mass index which were potential confounders. We also adjusted the analysis for clustering due to twins. The relative measures of effect on EBF in T-AGA, T- SGA, PT-AGA and PT-SGA were estimated and presented as forest plots. Data analysis was conducted with Stata version 16.0. (Stata Corp., College Station, TX, USA.

## Results

**Figure 1:**
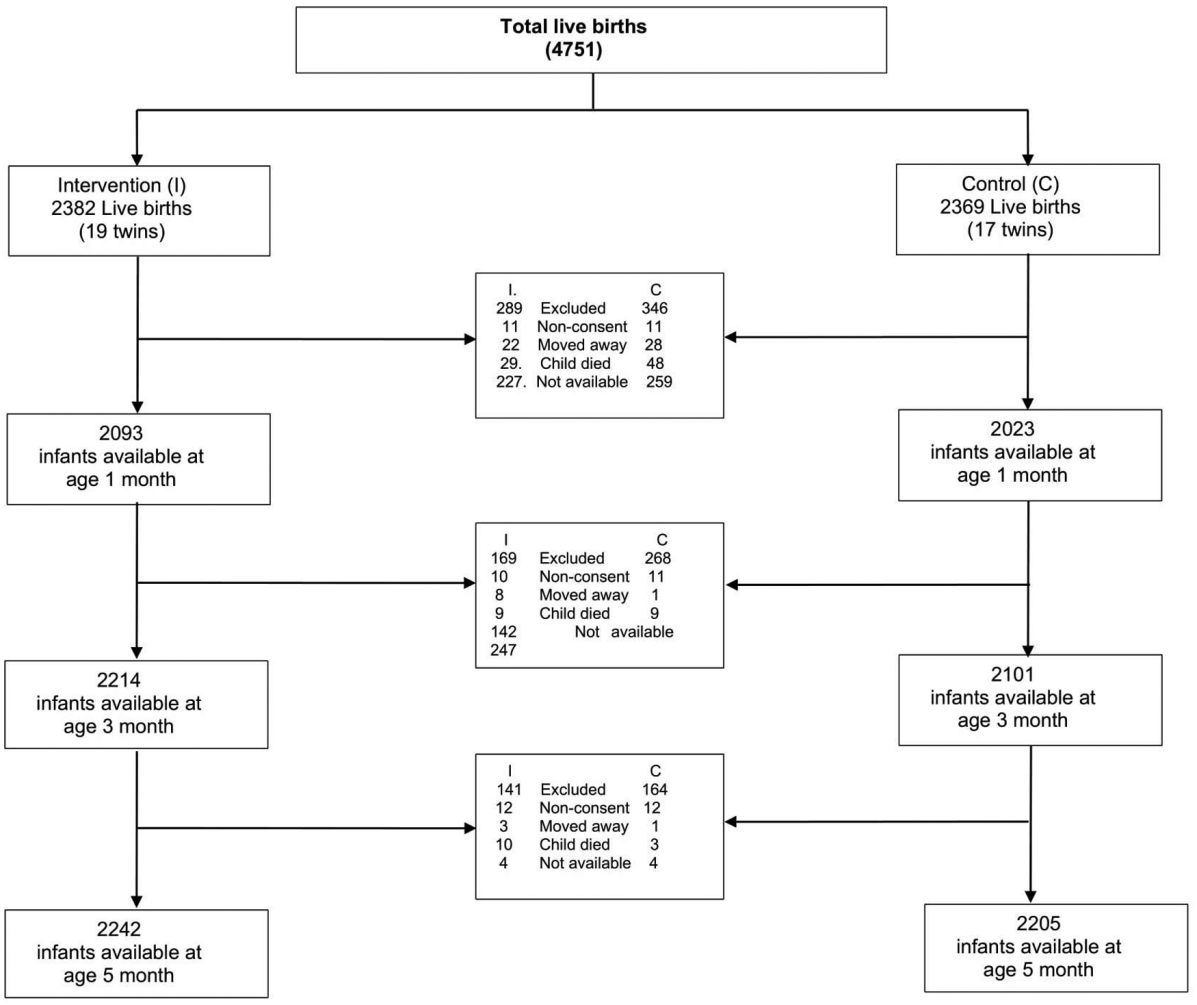
Flow Diagram.

In the analyses all live births were included at the baseline (Table 1). For describing the breastfeeding practices at different time points, a cross sectional approach was followed. Out of the total live births at baseline, the number of infants who had the information on breastfeeding practices at age 1, 3 and 5 months were selected for the analysis. Reasons for exclusions included family moved away, refused for interview, child died or were not available at the time of interview (Figure 1).

**Table 1:** Baseline characteristics.

	Intervention	Control
Infant characteristics	*N* = 2382	*N* = 2369
Female	1,139 (47.8)	1,189 (50.2)
Twins	17 (0.7)	17 (0.7)
Infant size and gestation at birth^a^	*N* = 2172	*N* = 2127
T-AGA	1,404 (64.64)	1,240 (58.30)
T- SGA	517 (23.80)	607 (28.54)
PT-AGA	171 (7.87)	196 (9.21)
PT-SGA	80 (3.7)	84 (3.95)
Maternal characteristics	*N* = 2365	*N* = 2352
Age (years), mean (SD)	23.77 (3.1)	23.81 (3.0)
Height (cm), mean (SD)	152.38 (5.7)	152.11 (5.6)
Height <150 cm	807 (34.1)	808 (34.4)
Women schooling ≥12 year	1,204 (50.9)	1,163 (49.5)
Homemaker	2,254 (95.3)	2,248 (95.6)
Underweight (<18.5 kg/m^2^)	356 (15.1)	347 (14.8)
Family had below poverty line card	82 (3.5)	98 (4.2)
Family covered by health insurance scheme	248 (10.5)	270 (11.5)
Joint or extended family^b^	1,579 (66.8)	1,511 (64.3)
Type of delivery	*N* = 2364	*N* = 2345
Caesarean	727 (30.8)	628 (26.8)
Normal Vaginal or Assisted vaginal (forceps or vacuum	1,637 (69.2)	1,717 (73.2)
Place of delivery	*N* = 2382	*N* = 2369
Large Hospital	1,681 (70.6)	841 (35.50)
Other hospitals or birthing centres	621 (26.0)	1,321 (55.8)
Home	80 (3.4)	207 (8.7)

Figures are number (percentages) unless stated otherwise.

^a^ There are 210 missing values for intervention group and 242 missing values for control group for infants whose gestational age or birth weight or both were not available; Term appropriate for gestational age (T-AGA): gestational age ≥37 weeks at birth and birth weight ≥10th centile to ≤90th centile as per Intergrowth standard; Term small for gestational age (T- SGA): gestational age ≥37 weeks at birth and birth weight <10th centile as per Intergrowth standard; Preterm appropriate for gestational age (PT-AGA): gestational age <37 weeks at birth and birth weight ≥10th centile to ≤90th centile as per Intergrowth standard; Preterm small for gestational age (PT-SGA): gestational age <37 weeks at birth and birth weight <10th centile as per Intergrowth standard.

SD, Standard deviation.

^b^ Joint or extended family: adult relatives other than enrolled woman’s husband and children living together in household.

Sociodemographic characteristics and infant details were represented as means (SD) or proportions as appropriate. The total number of live births were 2,382 in intervention group and 2,369 in control group. Baseline characteristics were comparable except for women’s height, proportion of women underweight, families possessing a below poverty line card, and place of birth (Table 1).

**Figure 2:**
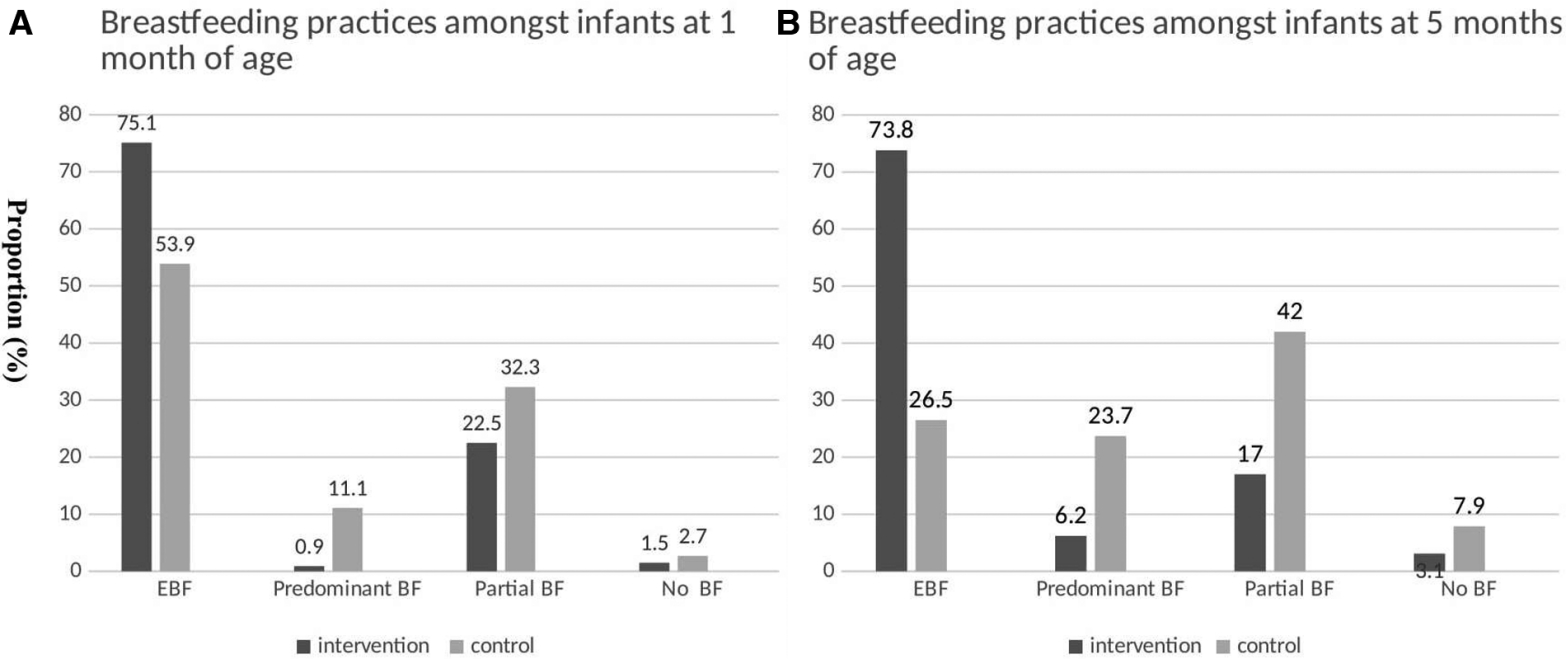
Breastfeeding practices amongst infants at age 1 month (**A**) and 5 months (**B**).

Mothers of 51.7% of the infants in intervention group initiated breastfeeding within the first hour of birth, in contrast to only 35.6% mothers in the control group. Breastfeeding practices of infants at 1 and 5 months of age are graphically represented in Figures 2A,B respectively. At 1 month of age, the rates of EBF was higher (75%), in the intervention group as compared to the control group (54%). Predominant breastfeeding (11.1%) and partial breastfeeding (32.2%) were higher in control group which received no breastfeeding counselling and support through the study, when compared with intervention group (Figure 2A).

At 5 months of age, EBF rate in the intervention group was sustained at 73.8% (Figure 2B). The rates of predominant (23.7%) and partial (42%) breastfeeding further increased in control group from the first month. EIBF which was 51% (IRR 1.38, 95% CI 1.28–1.48) in the intervention group compared to control group (35.6%) (Table 2). The proportion of exclusively breastfed infants at ages 1 month (IRR 1.37, 95% CI 1.28–1.48), 3 months (IRR 2.13, 95%CI 1.30–1.44) and 5 months (IRR 2.78, 95%CI 2.58–3.00) were higher in intervention as compared to the control group.

**Table 2:** Breastfeeding practices in all infants at infant age 1, 3 and 5 months.

Breastfeeding practices	Intervention	Control	Unadjusted IRR (95% CI)	Adjusted IRR^a^ (95% CI)
Early Initiation	1213/2345 (51.7)	820/2303 (35.6)	1.45 (1.36,1.55)	1.38 (1.28, 1.48)
Exclusive breastfeeding				
1 month−n/total (%)^a^	1592/2093 (76.1)	1111/2023 (54.9)	1.39 (1.32,1.45)	1.37 (1.30, 1.44)
3 month− /total (%)^a^	1950/2214 (88.1)	863/2101 (41.1)	2.14 (2.03, 2.26)	2.13 (2.02, 2.26)
5 months−n/total (%)^a^	1655/2242 (73.8)	585/2205 (26.5)	2.79 (2.59, 3.00)	2.78 (2.58, 3.00)

^a^Adjusted Incidence Rate Ratio (95% CI), adjusted for place of birth, family possesses below poverty line card, woman’s height, woman’s body mass index for potential confounder and twins for clustering within the household. Not corrected for multiple outcomes or comparisons.

**Figure 3:**
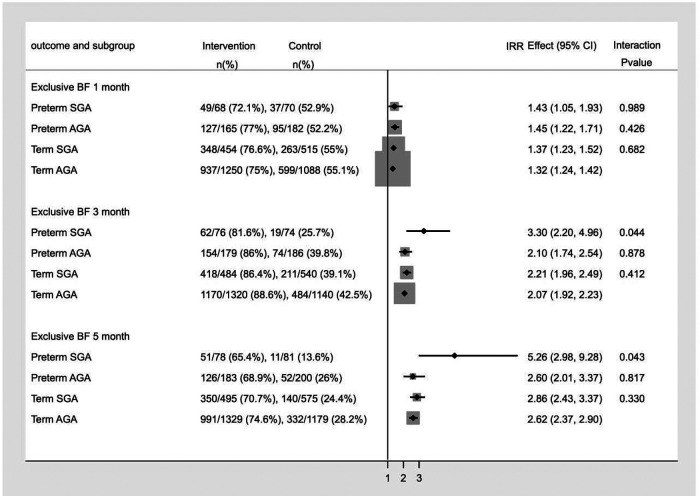
Subgroup analysis for Incidence of Exclusive breastfeeding in infants at 1, 3 and 5 months of age.

The relative measures of effect on breastfeeding practice, estimated in term appropriate for gestation age (T-AGA), term small for gestation age (T-SGA), preterm AGA (PT-AGA), preterm SGA (PT-SGA) infants are shown in Figure 3. We identified significant interaction (*p* value for interaction <0.05) between intervention and birth weight and gestational age at birth on exclusive breastfeeding at 3 and 5 months of age. Subgroup analysis showed that the impact of the intervention was greater on exclusive breastfeeding in PT- SGA infants at 3 months (IRR 3.30, 95% CI 2.20–4.96) and 5 months of age (IRR 5.26, 95% CI 2.98–9.28).

## Discussion

These analyses showed that provision of counselling and support to mothers for breastfeeding, improved practices on EIBF by 38% and lead to 2 times increase in practices of EBF in the first six months of life. The impact of this intervention was higher in preterm and SGA babies compared to other infants.

Similar findings have been observed from the evidence on breastfeeding counselling in LMIC’s. Interventions delivered concurrently in health facility and home settings lead to improvement in rates of EIBF (OR: 4.96; 95% CI: 2.88, 8.54) compared with interventions delivered individually at home or at health facility. For effect on EBF, pooled estimates showed that the odds of EBF at 1–5 months increased 3-fold (OR: 3.08; 95% CI: 2.57, 3.68) with only breastfeeding promotion interventions. This impact was highest when interventions were delivered in combination settings which was both in health facility and home (OR: 6.80; 95% CI: 3.75, 12.33) (25). Best outcomes are thus achieved when several interventions are delivered simultaneously and through multiple channels (26).

However, there is no population based evidence documenting effects of counselling on breastfeeding practices amongst infants based on gestational age and weight at birth from LMIC’s. It is widely known and accepted that small babies are at higher risk of non-exclusive breastfeeding due to factors like NICU admission, feeding intolerance and susceptibility to infections which are influenced by the gestational age at birth. NICU practices also play an important role in establishing the EBF in these babies, however most evidence is limited to high-income settings (27–29). In our study, while the infant was in hospital after birth, the hospital guidelines were followed for feeding as per treating physicians judgment. The study team only provided extra support to babies in intervention group for early initiation of breastfeeding and establishing breastfeeding. All our interventions were based on counselling about initiating breastfeeding and maintaining exclusive breastfeeding. All infants across both groups received standard of care at the health facility immediately post birth and intervention babies had no additional benefits at the hospital.

Through our study we have shown that through breastfeeding counselling centred around the small, at risk babies, the rates of EIBF and EBF can be improved which in turn will reduce the burden of morbidity and mortality in the first 6 months of life. This is the first study from India which has demonstrated the effects of breastfeeding counselling of mothers based on their infant’s gestational age and weight at birth. In addition, it reinforces that by providing breastfeeding counselling in both health facility and home settings and by offering hands-on support to mothers to tackle breastfeeding problems there were significant improvements in the breastfeeding rates of preterm and SGA babies, and improved adherence to exclusive breastfeeding in the first 6 months of life.

The strengths of the study include standardised outcome assessments, early pregnancy ultrasound based gestational age assessment, and generalisability to low and middle income urban populations.It is the first population-based studies from India where breastfeeding practices were ascertained in a randomized control trial based on gestational age at birth and birth weight of the infant.

The lockdowns due to the covid-19 pandemic affected intervention delivery which was one challenge we faced which may have affected outcome assessments for some infants. Restrictions in mobility made it difficult to conduct home visits for observing breastfeeding and to provide support and hands-on help to mothers. However, we overcame this through virtual contacts by lactation counsellors with the mothers.

### Policy implications

The key priority is support to small babies in breastfeeding right form birth and continued up to 6 months. This can only be achieved by early identification of these babies through Ultrasonography (USG) to ascertain accurate GA. The importance of EBF has been widely disseminated through various National programs, but the measures to be taken to improve the breastfeeding practices for vulnerable infants separately from the term and AGA infants needs to be defined. Special consideration should be given to understanding the barriers in implementation of breastfeeding interventions for these preterm and SGA babies. The availability of USG facility at different levels of the health systems to ascertain at risk infants (born preterm or SGA), though an expensive but one-time investment will improve lives of small babies by addressing the problems in breastfeeding in vulnerable infants right from birth.

## Conclusion

This is the first study where impact of breastfeeding counselling interventions in the first 6 months of life was assessed according to infant’s gestational age and weight at birth. The impact of this intervention was higher in preterm and SGA babies compared to other infants. In summary, the analyses show that breastfeeding counselling at both the health facility and at home can be translated to higher rates of EIBF and EBF, amongst the preterm and SGA babies thereby reducing adverse health outcomes during infancy and childhood. This is an important finding as preterm and SGA babies have a higher burden of mortality and morbidity during the first 6 months of life.

## Data Availability

The original contributions presented in the study are included in the article, further inquiries can be directed to the corresponding author.
